# Neuralgia and the Diseases That Resemble It

**Published:** 1872-03

**Authors:** 


*Neuralgia and the Diseases that resemble it.* By Francis E. Anstie, M. D., Lond. New York: D. Appleton & Co., 1872.

Dr. Anstie has produced a very valuable work on Neuralgia. In the introduction he speaks briefly of Pain in General and gives some very fine distinctions as to the varieties of pain and their causes. Closing his introductory remarks he gives the following distinctions:

1. Pain is not true hyperæsthesia; on the contrary, it involves a lowering of function.

2. Pain is due to a perturbation of nerve force, originating in dynamic disturbance either within or without the nervous system.

3. The susceptibility to this perturbation is great in proportion to the physical imperfection of the nervous tissue, *until* this imperfection reaches to the extent of cutting off communications, (paralysis.)

Part I—Is a very complete treatise on Neuralgia. Treating of its Clinical History, Complication, Pathology and Etiology, Diagnosis, Prognosis, and Treatment.

Part II—Treats at some length of the diseases that resemble Neuralgia, and is a valuable accompaniment of the first part. The work will, no doubt, hold a high position in medical literature.

